# Electroacupuncture and Transcutaneous Electrical Acupoint Stimulation for Perioperative Neurocognitive Disorder in Older Patients Undergoing Cardiac Surgery: Protocol for Systematic Review and Meta-Analysis

**DOI:** 10.2196/55996

**Published:** 2024-08-29

**Authors:** Yanbin Peng, Xuqiang Wei, Linxi Sun, Ke Wang, Jia Zhou

**Affiliations:** 1 Acupuncture Anesthesia Clinical Research Institute Yueyang Hospital of Integrated Traditional Chinese and Western Medicine Shanghai University of Traditional Chinese Medicine Shanghai China; 2 Office of National Clinical Research Base of TCM Yueyang Hospital of Integrated Traditional Chinese and Western Medicine Shanghai University of Traditional Chinese Medicine Shanghai China

**Keywords:** perioperative neurocognitive disorder, cardiac surgery, elderly patients, systematic review, meta-analysis

## Abstract

**Background:**

Perioperative neurocognitive disorder (PND) is a critical concern for older patients undergoing cardiac surgery, impacting cognitive function and quality of life. Electroacupuncture and transcutaneous electrical acupoint stimulation (TEAS) hold promise for mitigating PND. This protocol outlines a systematic review and meta-analysis to thoroughly assess the efficacy of electroacupuncture and TEAS in older patients undergoing cardiac surgery with PND, providing up-to-date evidence for PND prevention and treatment.

**Objective:**

This study aimed to thoroughly assess the efficacy of electroacupuncture and TEAS in older patients undergoing cardiac surgery with PND, providing up-to-date evidence for PND prevention and treatment.

**Methods:**

A comprehensive and systematic approach will be used to identify eligible studies from a diverse range of electronic databases, including 9 major sources such as PubMed (NLM) and Cochrane (Wiley), as well as 2 clinical trial registration websites. These studies will focus on investigating the effects of electroacupuncture and TEAS on PND in older patients undergoing cardiac surgery. The study selection will adhere to the criteria outlined in the patient, intervention, comparison, outcome, and studies (PICOS) format. Data extraction will be carried out by 2 independent researchers (YP and LS), using established tools to evaluate the risk of bias. The primary outcome will be PND incidence, with secondary outcomes including Mini Mental State Examination scores, neuron-specific enolase, S100β, interleukin-1β, interleukin-6, tumor necrosis factor-α, time to first flatus, first defecation, bowel sound recovery, and hospitalization duration to be selectively reported. Adverse events linked to acupuncture, such as bleeding, needle site pain, and local reactions, rather than serious adverse events, will also be considered. Meta-analysis will be performed using appropriate statistical methods to assess the overall effect of electroacupuncture and TEAS on PND prevention, treatment, or other relevant outcomes. The Cochrane Collaboration Risk of Bias tool will be used for assessment, and data synthesis will be executed using the RevMan 5.4 software (Cochrane).

**Results:**

We plan to summarize the eligible studies through the use of a PRISMA (Preferred Reporting Items for Systematic Reviews and Meta-Analyses) flowchart. The findings will be showcased in the form of a summary table of evidence. Figures and forest plots will be used to illustrate the outcomes of the meta-analysis.

**Conclusions:**

The impacts of electroacupuncture and TEAS interventions on PND in older patients undergoing cardiac surgery have not yet been established. This protocol addresses a critical gap by thoroughly assessing electroacupuncture and TEAS for PND in older patients undergoing cardiac surgery, enhancing understanding of nonpharmacological interventions, and guiding future research and clinical practices in this field. Its strength lies in rigorous methodology, including comprehensive search strategies, independent review processes, and thorough assessments of the risk of bias.

**Trial Registration:**

PROSPERO CRD42023411927; https://tinyurl.com/39xdz6jb

**International Registered Report Identifier (IRRID):**

PRR1-10.2196/55996

## Introduction

### Perioperative Neurocognitive Disorder

Perioperative Neurocognitive Disorder (PND) remains a significant concern in the context of cardiac surgery [1]. PND encompasses a spectrum of cognitive deficits that manifest during the perioperative period, ranging from mild cognitive impairment to delirium and overt dementia [2]. These deficits impact a range of cognitive areas, encompassing memory, concentration, executive skills, and language abilities [3,4]. The manifestation of these impairments becomes evident in the aftermath of surgery [5], typically peaking within the initial postoperative week and occasionally enduring for an extended duration [3,6]. These cognitive changes carry substantial implications for patients’ quality of life, functional independence, health care expenditure, and postoperative outcomes [7-9].

### PND in Older Patients Undergoing Cardiac Surgery

The relationship between PND and older patients undergoing cardiac surgery is of particular interest, given the vulnerability of this population to cognitive decline [10,11]. As the worldwide population ages and surgical methods and anesthetic practices advance, the volume of individuals undergoing cardiac surgery is on the rise, prompting a greater focus on enhancing their perioperative management. The exact pathophysiological mechanisms of PND are multifactorial and complex, remaining elusive. Factors such as age-related physiological changes, surgical anesthesia stress, increased susceptibility to inflammation, and potential pre-existing cognitive deficits may render older patients more susceptible to PND [12-19]. Furthermore, cardiac surgery introduces potential risks of neurocognitive complications due to cardiopulmonary bypass and associated factors [20,21]. Epidemiological studies have indicated that the occurrence rates of PND in older individuals undergoing cardiac surgery vary between 25% and 50% [22-25]. This wide variability underscores the complexity of PND’s etiology and the need for comprehensive investigation and intervention strategies [26].

### Related Management Methods of PND

Current conventional approaches for managing PND primarily focus on mitigating risk factors through neuroprotective agents, refined anesthesia management, and inflammation minimization [16,27]. Nevertheless, these strategies have limitations in preventing PND occurrence or effectively treating established cases, and many medications exhibit side effects, falling short of addressing the disorder’s multifaceted nature and potential adverse effects [28]. To bridge these gaps in the treatment landscape, exploring alternative or supplementary therapeutic interventions targeting PND’s multifaceted nature is compelling to attenuate cognitive decline. Among emerging alternative approaches, acupuncture has garnered attention as a potentially promising intervention for PND. Rooted in traditional Chinese medicine, acupuncture is suggested to offer advantages such as modulating neuroinflammation, enhancing cerebral perfusion, and promoting neural plasticity [29-35]. Furthermore, acupuncture’s favorable safety profile aligns with the older adult population’s increased susceptibility to pharmacological agents’ adverse effects [36]. Particularly, electroacupuncture and transcutaneous electrical acupoint stimulation (TEAS), involving noninvasive application of electrical currents to modulate neural activity, hold promise as nonpharmacological interventions that may prevent or ameliorate cognitive decline. The results of a randomized controlled trial (RCT) revealed that the acupuncture group exhibited a shorter time to first remission of delirium and a significantly higher number of delirium-free days compared with the standard treatment group in the treatment of older patients with delirium, with no observed adverse safety events [37].

### Limitations of Previous Studies

While previous studies have explored various interventions for PND, previous meta-analyses have primarily focused on the overall population without distinguishing between surgical types, resulting in considerable heterogeneity [38,39]. Currently, there is a recognized gap in the literature regarding the impact of electroacupuncture and TEAS, specifically on PND in older patients undergoing cardiac surgery, with no systematic reviews or meta-analyses available. A previous meta-analysis included 18 RCTs to evaluate the impact of TEAS on cognitive function in older individuals after general anesthesia. Despite this effort, it was not possible to analyze the incidence of postoperative delirium due to the limited number of original studies and poor methodology [40]. In 2021, a review of 16 RCTs involving 1241 patients suggested that acupuncture appears to be a promising adjunct intervention for the treatment and prevention of postoperative cognitive dysfunction. However, the evidence is insufficient and has limitations [41]. Another systematic review and meta-analysis in 2023 included 12 studies with a total of 1058 patients to explore the use of acupuncture-related techniques in treating postoperative cognitive complications. Nonetheless, the enrolled studies varied in surgical types, and no definitive conclusions could be drawn [42]. Consequently, the acupuncture research landscape for PND remains intricate with varied study methodologies, inconsistent intervention protocols, and divergent outcome measures contributing to fragmented evidence, lacking a comprehensive synthesis. The curative effect of acupuncture on PND remains controversial, and the optimal acupuncture regimen for preventing or treating PND is unclear.

### Aims

Given the gaps in knowledge and potential implications for clinical practice, this systematic review and meta-analysis protocol aims to consolidate current research findings on electroacupuncture and TEAS interventions’ potential effectiveness in preventing or ameliorating PND in older patients undergoing cardiac surgery. By synthesizing available evidence, we endeavor to provide a comprehensive overview of the existing literature, identify key research gaps, and offer insights into potential mechanisms. Specifically, our objective is to offer a comprehensive perspective by comparing treatment outcomes and phenomena associated with acupuncture and addressing the questions, such as (1) what is the impact of electroacupuncture and TEAS on the incidence and severity of PND in older patients undergoing cardiac surgery? (2) What are the effects of electroacupuncture and TEAS on serum biomarkers in older patients undergoing cardiac surgery? Through addressing current knowledge gaps, these insights have the potential to inform clinical practice, clarify controversies, and shape future research endeavors. In addition, they contribute to the development of evidence-based strategies aiming to optimize perioperative care for older patients undergoing cardiac surgery, ultimately leading to improvements in postoperative cognitive outcomes.

## Methods

### Study Registration

The meta-analysis protocol has been registered with PROSPERO (International Prospective Register of Systematic Reviews; registration CRD42023411927). In addition, the PRISMA (Preferred Reporting Items for Systematic Reviews and Meta-Analyses) protocol guidelines have been used to report this protocol (Multimedia Appendix 1) [43]. Our systematic review will be conducted following the guidelines outlined in the Cochrane Collaboration Handbook [44], and we will ensure transparency in reporting by adhering to the PRISMA 2020 guidelines [45].

### Study Design

We intend to incorporate a range of study designs, such as RCTs, quasi-experimental approaches, and prospective cohort studies, as well as controlled clinical trials, that assess the effectiveness and safety of electroacupuncture and TEAS in PND in older patients undergoing cardiac surgery [46].

### Type of Participants

Incorporating patients who are 60 years of age or older, this study endeavors to investigate the inclusion of individuals scheduled for elective cardiac surgery, ensuring that they do not exhibit any preexisting neurocognitive disorders. It is worth noting that participants’ eligibility will not be restricted by factors such as gender, race, surgical history, or underlying medical conditions.

### Type of Intervention

The treatment groups will undergo electroacupuncture and TEAS therapies. The study will not impose any limitations on the sample size, intervention duration, perioperative care, or the underlying treatment modalities used. This inclusivity will facilitate a comprehensive exploration of the treatment’s efficacy.

### Type of Control Groups

To provide a comparative framework, the control groups may encompass a range of approaches. These include the application of general anesthesia as part of conventional care, sham acupuncture, as well as pharmacotherapy involving Western medicine. It is crucial to note that interventions deviating from these specified approaches will be excluded from the comparator groups, ensuring a coherent and consistent comparison.

### Types of Outcome Measures

To assess the impact of the chosen interventions, a comprehensive set of outcomes has been strategically determined. Primary outcomes will focus on evaluating the incidence of PND. Emphasizing the significance of a comprehensive evaluation, a host of secondary outcomes will be collected and subjected to meticulous analysis. These encompass evaluating neurocognitive function through the use of Mini Mental State Examination (MMSE) scores, alongside measuring serum levels of neuron-specific enolase, S100β, interleukin-1β, interleukin-6, and tumor necrosis factor-α. In addition, vital secondary outcomes include evaluating the time to first flatus, first defecation, bowel sound recovery, and the duration of hospitalization. It is crucial to highlight that adverse events associated with acupuncture, such as bleeding, needle site pain, and other localized reactions will be documented and analyzed, while serious adverse events will be addressed separately [47].

### Search Strategy

**Figure 1:**
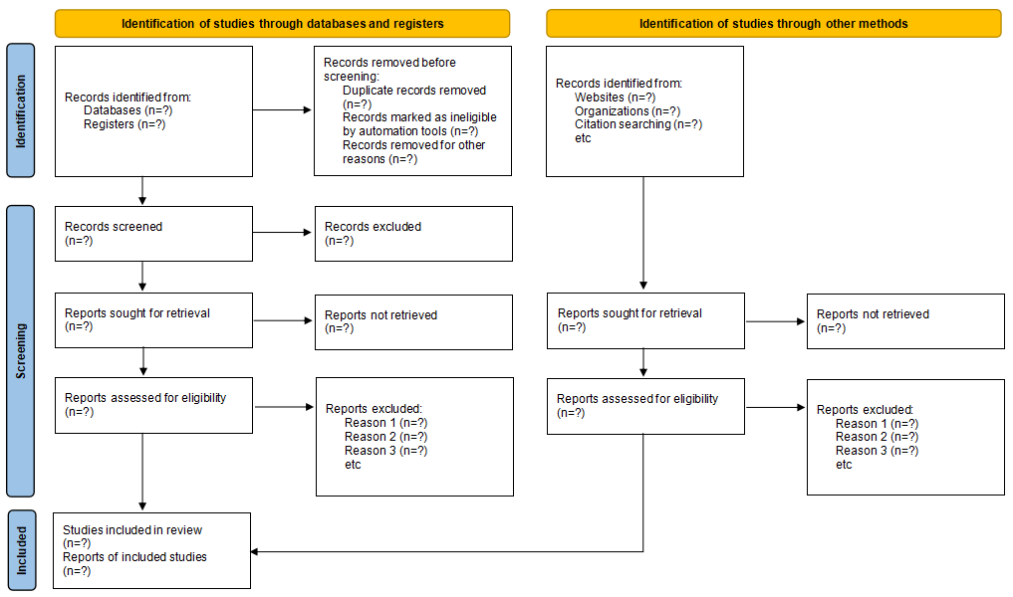
PRISMA flow diagram of study identification and selection. PRISMA: Preferred Reporting Items for Systematic Reviews and Meta-Analyses [43].

To perform a thorough review of the literature, we have formulated an expansive search approach, encompassing the exploration of 9 distinct databases, specifically PubMed (NLM), Embase (Elsevier), Cochrane Library (Wiley), Web of Science (Clarivate), Scopus (Elsevier), China National Knowledge Infrastructure (Tongfang Knowledge Network Technology), Chinese Biomedical Literature Database (SinoMed), Wan Fang Database, and VIP Database. In addition, 2 registered websites [48,49] will also be explored. To maximize the scope of the review, reference lists from relevant articles will be manually examined. Importantly, there will be no limitations placed on the publication status, source country, or publication year. To ensure a comprehensive search, a predefined list of search terms, including “electroacupuncture,” “TEAS,” “PND,” ‘cardiac surgery,” “elderly,” and “RCTs,” will be used in combination with the subject words unique to each database [50]. Notably, the comprehensive search approach used within PubMed can be found in Table 1, with specific search strategies tailored for individual databases accessible in the Multimedia Appendix 2. To further enhance this comprehensive approach, additional sources, such as dissertations, conference papers, gray literature, and unpublished research from relevant entities, will also be assessed and advice will be sought from experts in acupuncture and cardiac surgery. To streamline this process, EndNote 20 (Clarivate) will be used to manage the literature and eliminate any duplicate studies. In order to uphold research integrity, the eligibility of studies will be independently screened and evaluated by 2 researchers, YP and LS. Any disagreements will be resolved through deliberation with other members of the research team. Both Chinese and English language studies will be retrieved and considered in this study. The manuscript screening process is shown in Figure 1.

**Table 1:** The search strategy for PubMed.

Order	Strategy
#1	“Postoperative Cognitive Complications” [MeSH^a^]
#2	““postoperative delirium” [MeSH]
#3	“”perioperative neurocognitive disorder” [Title/Abstract]
#4	““cognit*^b^” [Title/Abstract] OR “cognition disorder” [Title/Abstract] OR “”cognition impairment” [Title/Abstract] OR ““cognition decline” [Title/Abstract] OR “cognitive dysfunction” [Title/Abstract] OR “”cognitive function” [Title/Abstract] OR “delirium” [Title/Abstract] OR “neurocognitive disorder” [Title/Abstract]
#5	“postop*^b^” [Title/Abstract] OR “postoperative*^b^” [Title/Abstract] OR ““postoperative” [Title/Abstract] OR “postoperative period” [Title/Abstract]
#6	#4 AND #5
#7	#1 OR #2 OR #3 OR #6
#8	“acupuncture” [MeSH Terms] OR “acupuncture therapy” [MeSH Terms] OR “acupuncture therapy” [MeSH Terms] OR “electroacupuncture” [MeSH Terms]
#9	“Acupuncture” [Title/Abstract] OR “Electroacupuncture” [Title/Abstract] OR “EA^c^” [Title/Abstract] OR “transcutaneous electrical acupoint stimulation” [Title/Abstract] OR “TEAS^d^” [Title/Abstract]
#10	#8 OR #9
#11	((((((((((((Thoracic Surgery [MeSH Terms]) OR (Cardiac Surgical Procedures [MeSH Terms])) OR (Surgery, Thoracic)) OR (Surgery, Cardiac)) OR (Surgery, Heart)) OR (Heart Surgery)) OR (Cardiac Surgery)) OR (Procedure*^b^, Cardiac Surgical)) OR (Surgical Procedure*^b^, Cardiac)) OR (Surgical Procedure*^b^, Heart)) OR (Cardiac Surgical Procedure*^b^)) OR (Heart Surgical Procedure*^b^)) OR (Procedure*^b^, Heart Surgical)
#12	elderly OR older OR geriatric
#13	#7 AND #10 AND #11 AND #12

^a^MeSH: Medical Subject Headings.

^b^*: special character that represents zero or more characters in wildcard searching.

^c^EA: electroacupuncture.

^d^TEAS: transcutaneous electrical acupoint stimulation.

### Inclusion and Exclusion Criteria

The criteria for inclusion in this review were established following the patient, intervention, comparison, outcome, and studies (PICOS) framework (Textbox 1). There were no language restrictions and the publication date considered was up to June 15, 2024.

**Inclusion and exclusion criteria.**

**Inclusion criteria:**

- The study’s target population comprises older individuals (aged 65 years or older) undergoing cardiac surgery. These patients may exhibit a range of cardiac conditions necessitating surgical procedures, including coronary artery bypass grafting, valve replacement, or a combination of interventions.
- The primary intervention of interest is electroacupuncture or transcutaneous electrical acupoint stimulation (TEAS). Studies using either of these interventions as a therapeutic modality in the perioperative period will be considered. The interventions may involve varying frequencies, intensity, waveform, durations, and acupoint selections. Research that involves other forms of acupuncture or nonacupuncture interventions will be excluded unless they are used within the context of a comparative group.
- Eligible studies will encompass those incorporating an appropriate comparative group. This may involve sham or placebo interventions, standard care lacking any electroacupuncture and TEAS application, or alternative interventions designed to mitigate perioperative neurocognitive disorder. Studies comparing different forms of electroacupuncture and TEAS protocols will also be considered, including studies comparing different frequencies, intensities, waveforms, acupoints, or durations of electroacupuncture and TEAS interventions.
- Primary outcome: incidence of perioperative neurocognitive disorder. Secondary outcomes: the Mini Mental State Examination scores, neuron-specific enolase, S100β, interleukin-1β, interleukin-6, and tumor necrosis factor-α, time to first flatus, first defecation, bowel sound recovery, and duration of hospitalization will be selectively reported. Adverse incidents linked to acupuncture, such as bleeding, discomfort at needle insertion sites, and local reactions, will be considered, with a focus on non-serious adverse events.
- The eligible studies will encompass randomized controlled trials, quasi-experimental designs, prospective cohort studies, as well as controlled clinical trials.

**Exclusion criteria:**

- Research focusing on patients who are not older adults (aged <65 years) or those undergoing surgeries unrelated to cardiac procedures.
- Studies lacking proper cognitive assessment as an outcome measure.
- Studies involving animals, retrospective investigations, case reports, commentaries, literature reviews, duplicate publications, and articles with unavailable full text.
- Studies where electroacupuncture and TEAS is combined with other interventions that cannot be adequately isolated for analysis.

### Data Extraction and Risk of Bias Assessment

The extraction of data will be carried out independently by 2 researchers (YP and LS). Throughout the process, any differences will be resolved through discussion or by involving another researcher (KW). The essential data extracted from the papers include the author’s name, publication year, country, sample size, gender distribution, age distribution, disease duration, treatment duration, intervention details, and study outcomes. The inclusion or exclusion of electroacupuncture and TEAS interventions in each article will be itemized meticulously in accordance with the STRICTA (Standards for Reporting Interventions in Clinical Trials of Acupuncture) checklist [51,52]. A predesigned data collection form will be used to conduct data extraction for each study, with a prime focus on extracting the PICOS components and funding details.

A total of 2 evaluators (YP and LS) will individually evaluate potential biases in each study by applying the criteria specified in the Cochrane Handbook for Systematic Reviews of Interventions. Any discrepancies will be resolved through constructive dialogue or by enlisting the participation of another author (KW). We will assess the literature’s quality using the “bias risk assessment tool” recommended by the Cochrane Collaboration. The bias risk will be documented in a table, where high risk will be indicated in red, low risk in green, and uncertain risk in yellow. We will add annotations to the tables to supplement information about the risk of bias and unpublished data sources obtained by contacting trial authors. Each item in the risk of bias assessment will be considered independently without assigning an overall score. We will consider the potential impact of bias in each study when assessing the effectiveness of the treatment, as it could affect the results.

### Data Synthesis and Analysis

We will use RevMan 5.4 software to conduct the meta-analysis and generate the risk of bias graph. For binary data, we will use risk ratios and Mantel-Haenszel tests, along with a 95% CI analysis. Continuous variables will be represented as the mean difference, effect value, and 95% CI. In cases where outcome measures vary across studies, the mean SD will be used. To assess statistical heterogeneity, we will perform tests including the *I*^2^ statistic, which ranges from 0% to 100%. Statistical heterogeneity will be evaluated using the standard chi-square test (α=.1) and the *І*^2^ test [53]. If *І*^2^≤50%, the fixed-effects model will be used. If *І*^2^>50%, the random-effects model will be applied [54]. In instances of conspicuous clinical heterogeneity, the random-effects model will be used. The forest plots will display the results of the meta-analysis, where a *P* less than .05 indicates statistical significance. If a study is not amenable to quantitative synthesis, a descriptive analysis will be conducted and presented using a summary table.

### Management of Missing Data

Efforts will be made to reach out to the primary author or corresponding author of the initial publication for the acquisition of any absent or inadequate data. In cases where such data cannot be obtained, an examination of the available data will be performed.

### Subgroup and Sensitivity Analyses

If excessive heterogeneity is observed, we will conduct grouping and sensitivity analyses to investigate the potential sources of major inconsistencies or heterogeneity. Subgroup analysis will be performed based on different types of acupuncture and PND. The type of acupuncture, surgical methods, anesthesia, and other complicating factors will be analyzed if there are a sufficient number of studies and a variety of regimens are used [47]. During the sensitivity analysis, certain trials will be omitted to pinpoint potential origins of bias and evaluate the uniformity of the meta-analysis findings. Specifically, studies that did not report an intention-to-treat analysis, experienced high rates of participant dropout, or had other missing data will be excluded [55]. In addition, sensitivity analysis will be performed to evaluate the potential influence of any missing data on the outcomes.

### Evaluation of Publication Bias

In cases where there are more than 10 studies included for each outcome, we will use funnel plots generated in RevMan to visually assess the presence of publication bias and assess the quality of evidence.

### Grading of Recommendations Assessment, Development, and Evaluation Quality of Evidence

We will use the Grading of Recommendations Assessment, Development, and Evaluation criteria to rank the quality of evidence. We will then evaluate the level of evidence and strength of recommendations for the outcomes under consideration. The levels of evidence certainty encompass 4 categories, that are “high,” “moderate,” “low,” or “very low.” These categories signify the degree of confidence in the effect estimate, with “high certainty” denoting a robust level of confidence and “very low certainty” indicating minimal confidence [56]. Confidence levels may be adjusted based on considerations such as risk of bias, imprecision, inconsistency, indirectness, or publication bias. Since only RCTs will be included in the review, reasons for updating the study (eg, large effects, dose-response relationships, and confounders) do not apply [57].

### Sample Size Evaluation

Trial sequential analysis (TSA) will be used for sample size estimation in systematic reviews or meta-analyses. TSA overcomes the limitations of classical systematic reviews or meta-analyses [58]. It reduces the occurrence of incorrect positive outcomes resulting from random errors in situations where the number of cases included in a meta-analysis is insufficient. TSA refers to the required information size, which denotes the minimum number of cases necessary to achieve statistically significant distinctions in a meta-analysis. Generally, the sample size requisite for meta-analysis is regarded as no smaller than what is needed for a properly designed and statistically robust individual RCT. In this study, the TSA program version 0.9.5.10 Beta (Copenhagen Trial Unit) will be used to control the risks of type I and type II errors by estimating RIS (required information size) and monitoring testing sequences [59].

### Patient and Public Involvement

The design, execution, reporting, and dissemination plans of this research will not involve the participation of patients and the public, given the nature of the systematic review and meta-analysis. Nevertheless, upon completion of the research, our findings will be disseminated and shared through peer-reviewed journals, conferences, and seminars focusing on the care and treatment of older patients undergoing surgery.

### Ethical Considerations

Ethical clearance is not required for this systematic review, as it relies on previously published data. Because no original data will be collected, this study does not require ethical approval. The results will be presented at conferences and published in journals with peer review.

### Validity, Reliability, and Rigor

Our systematic review will present findings in accordance with the best practice PRISMA guidelines, and meta-analyses will be carried out using a random-effects meta-analytic approach, as recommended for synthesizing study outcomes [60].

### Amendments

The protocol for this systematic review will be amended when necessary.

## Results

The results of this study will be presented at conferences and published in journals with peer review. We will maintain regular updates to our searches across all databases every 2 months to incorporate any newly available data into the systematic review.

## Discussion

### Hypothesized

Due to increasing cardiovascular issues, there is a growing demand for cardiac surgeries [61]. In accordance with traditional Chinese medical principles, which emphasize the interconnectedness of the heart and the brain, it is suggested that the “heart and brain co-govern spirit,” wherein the spirit encompasses both the nervous system and mental faculties. Consequently, patients with cardiac surgery are more susceptible to cognitive impairment [62,63]. Research has indicated that reduced cardiac ejection fraction can activate the autonomic nervous system, elevate catecholamine and endothelin levels, disrupt cerebral autoregulation, and subsequently cause insufficient cerebral perfusion, diminished cerebral blood flow, damage to nerve cells, onset of cognitive deficiencies, and changes in mental conditions. These factors align with some of the underlying factors associated with acupuncture [64]. Our study hypothesizes that electroacupuncture and TEAS can significantly reduce the incidence of PND in older patients undergoing cardiac surgery.

### Potential Effect of Acupuncture on PND

To date, no interventions are offering a complete cure for PND, and common pharmacotherapies may pose challenges due to patient compliance and potential adverse reactions [65,66]. Measures aimed at enhancing patients’ understanding and acceptance of the disease and its treatment, such as electroacupuncture and TEAS, have gained prominence in recent years. These simple, convenient, cost-effective, and safe modalities empower patients to proactively monitor and manage PND. The empirical use of acupuncture in treating cognitive impairments spans millennia [28]. Previous research suggests that acupuncture may serve as an effective adjunctive therapy for neurological conditions such as depressive symptoms [67], mild cognitive impairment [68], poststroke cognitive impairment [69,70], vascular dementia [71], schizophrenia [72] and Alzheimer disease [73]. Electroacupuncture and TEAS represent novel acupuncture therapies that combine electrical stimulation with acupuncture point stimulation. As nonpharmacological interventions, they offer advantages such as there are no drug-related side effects and minimal invasiveness, making them widely used in clinical practice, especially in perioperative management [74-76]. Numerous emerging trials presently demonstrate the clinical efficacy of electroacupuncture and TEAS in managing PND [38,39,68,77]. Currently, theories related to the pathogenesis of PND include central inflammatory responses, reduced central cholinergic system function, synaptic dysfunction, abnormal protein function, and disturbances in gut microbiota [78,79]. Among these, the central inflammatory response mechanism is of particular concern [80-82]. Early studies have suggested that electroacupuncture and TEAS can alleviate both central and peripheral inflammatory responses, inhibit microglial cell activation, suppress neuronal apoptosis, and provide significant neuroprotection through various pathways, thereby delaying the pathological progression of PND [83,84]. Furthermore, numerous emerging trials have also demonstrated that adjunctive use of electroacupuncture and TEAS in the perioperative period has regulatory effects on the gastrointestinal system, improves circulation, reduces anesthesia requirements, enhances immunity, decreases inflammation, and alleviates stress. This leads to shorter patient recovery and hospitalization times, ultimately improving patient quality of life and indicating the potential efficacy of electroacupuncture and TEAS in treating PND [85,86]. Regrettably, despite certain systematic reviews having assessed the effectiveness of electroacupuncture and TEAS-related approaches in PND management, these reviews invariably manifest limitations encompassing diversities in terms of race, age, gender, intervention methodologies, and acupuncture treatment protocols. Such diversification might engender heightened clinical and statistical heterogeneity [87]. In addition, some of the earlier studies lacked standardized protocols for assessing PND, leading to inconsistencies in the definition and diagnosis of the condition. These constraints have posed challenges in developing an extensive comprehension of the efficacy of approaches such as electroacupuncture and TEAS in preventing or mitigating PND. Therefore, as of the present, no compelling evidence has materialized, thereby limiting the applicability of acupuncture.

### Outcome Indicator Selection Reasons

In light of the limitations in previous research, our meta-analysis concentrates on a specific aspect—the impact of electroacupuncture and TEAS on PND in older patients undergoing cardiac surgery. By narrowing our scope to this particular intervention and patient population, we aimed to provide a more comprehensive and normative approach to evaluating the evidence for electroacupuncture and TEAS in preventing or treating PND. This approach allows for a more homogeneous pool of studies, which enhances the comparability and generalizability of our findings. The primary outcome measure (ie, the incidence of PND), was chosen as it represents the most direct and fundamental outcome of cognitive dysfunction postcardiac surgery. It facilitates an assessment of whether electroacupuncture and TEAS have the potential to reduce the risk of cognitive impairment. The inclusion of the MMSE as a secondary outcome measurement is motivated by its widespread use as a highly sensitive, standardized, and reliable tool for assessing cognitive function. MMSE scores will enable a detailed examination of specific cognitive domains, allowing for a more nuanced analysis of the impact of electroacupuncture and TEAS on cognitive performance. Neurobiological markers such as neuron-specific enolase, S100β, and proinflammatory cytokines including interleukin-1β, interleukin-6, and tumor necrosis factor-α are selected as secondary outcomes to explore the potential mechanisms underlying observed cognitive effects. Monitoring these biomarkers will provide insights into the neuroinflammatory response and neuronal damage associated with PND, helping to elucidate the pathways through which electroacupuncture and TEAS may exert their influence. In addition to cognitive outcomes, we are also attentive to impacts on certain clinical manifestations, with gastrointestinal function being a crucial aspect of overall recovery after cardiac surgery. This study will assess postoperative parameters of gastrointestinal recovery, including the first flatus time, the first defecation time, and the recovery of bowel sounds. Electroacupuncture and TEAS are considered to potentially accelerate intestinal function recovery through their effects on the neuroendocrine and autonomic nervous systems, thereby influencing these indicators. These measures offer a comprehensive perspective on the impact of acupuncture-based interventions on postoperative physiological recovery, potentially influencing patient comfort and overall surgical outcomes. Furthermore, prolonged hospitalization is associated with increased medical costs and potential complications. Evaluating the impact of electroacupuncture and TEAS on the length of hospital stay provides valuable insights into the overall recovery trajectory and cost-effectiveness of the intervention. To summarize, the selection of these primary and secondary outcome measures was driven by the need to capture a holistic picture of the intervention’s multifaceted impact on cognitive function, neuroprotection, inflammatory response, and overall postoperative recovery in older patients undergoing cardiac surgery. These outcomes collectively aim to contribute to the existing knowledge base, provide scientific information for clinical practice, and potentially improve patient outcomes in this vulnerable population.

### Implications and Conclusions

If our meta-analysis can demonstrate the effectiveness of electroacupuncture and TEAS in preventing or ameliorating PND in older patients undergoing cardiac surgery, its significance will be profound. Firstly, patients undergoing cardiac surgery might experience better cognitive results and an improved quality of life postoperatively. This finding offers significant hope for older patients who are particularly prone to PND. Frontline health care providers, including cardiac surgeons and anesthesiologists, can benefit from our research results by considering the inclusion of electroacupuncture and TEAS in their perioperative care protocols. This may result in enhanced patient results, diminished health care expenses linked to the treatment of complications related to PND, and increased patient contentment. In addition, our research may inspire additional exploration in the realm of safeguarding cognitive function during the perioperative period. It may encourage researchers to explore other complementary interventions, refine protocols, and conduct RCTs to establish causality and optimize clinical practices. In conclusion, our meta-analysis represents a critical step toward addressing the limitations of previous research on PND in older patients undergoing cardiac surgery.

### Limitations

It is essential to acknowledge the limitations of the proposed systematic review and meta-analysis. Variability in acupoint selection and the heterogeneity of cardiac surgical procedures may introduce challenges in synthesizing the evidence. In addition, publication bias and the quality of included studies may impact the robustness of the conclusions drawn. Building upon the results of this protocol, future research avenues could explore the optimal timing, duration, and frequency of electroacupuncture and TEAS interventions. Moreover, investigating the mechanisms underlying the observed effects may provide valuable insights into the neurobiological processes influenced by acupuncture-based therapies. To conclude, by focusing on electroacupuncture and TEAS and adopting a more standardized approach, we hope to contribute valuable insights for clinicians and researchers, positioning acupuncture as a viable treatment option for PND. This, in turn, can provide better choices for patient management and guide future research efforts to improve perioperative cognitive outcomes.

## Appendix

Multimedia Appendix 1 PRISMA-P (Preferred Reporting Items for Systematic Reviews and Meta-Analyses Protocols) 2015 checklist.

Multimedia Appendix 2 Search strategy.

## Abbreviations

MMSE: Mini Mental State Examination
PICOS: patient, intervention, comparison, outcome, and studies
PND: Perioperative Neurocognitive Disorder
PRISMA: Preferred Reporting Items for Systematic Reviews and Meta-Analyses
PROSPERO: International Prospective Register of Systematic Reviews
RCT: randomized controlled trial
STRICTA: Standards for Reporting Interventions in Clinical Trials of Acupuncture
TEAS: transcutaneous electrical acupoint stimulation
TSA: trial sequential analysis

## Data Availability

The data sets generated during and/or analyzed during this study are available from the corresponding author on reasonable request.
