# Supplementary material for: Electroacupuncture and Transcutaneous Electrical Acupoint Stimulation for Perioperative Neurocognitive Disorder in Older Patients Undergoing Cardiac Surgery: Protocol for Systematic Review and Meta-Analysis
Source: JMIR Res Protoc. 2024 Aug 29;13:e55996. doi: 10.2196/55996 (PMC11393506; doi:10.2196/55996)
Supplement: Multimedia Appendix 2 [file resprot_v13i1e55996_app2.docx]

**Table S1** The search strategy for Web of Science

| Order | Strategy |
| --- | --- |
| #1 | **TS=(**cognit* OR "cognition disorder" OR "cognition impairment" OR "cognition decline" OR "cognitive dysfunction" OR "cognitive function" OR "delirium" OR "neurocognitive disorder" OR "delayed neurocognitive recovery" OR "neurocognitive abnormalities"**)** |
| #2 | **TS=(**postop* OR "postoperative*" OR "perioperative" OR "postoperative period") |
| #3 | **TS=(**acupuncture OR electroacupuncture OR "EA" OR "transcutaneous electrical acupoint stimulation" OR "transcutaneous electrical stimulation" OR "transcutaneous electrical nerve stimulation" OR "TEAS") |
| #4 | **TS=(random OR randomization OR randomized OR randomised OR randomly)** |
| #5 | **#1 AND #2 AND #3 AND #4** |
| #6 | Thoracic Surgery (All Fields) or Surgery, Thoracic (All Fields) or Surgery, Cardiac (All Fields) or Surgery, Heart (All Fields) or Heart Surgery (All Fields) or Cardiac Surgery (All Fields) or Cardiac Surgical Procedures (All Fields) or Procedure*, Cardiac Surgical (All Fields) or Surgical Procedure*, Cardiac (All Fields) or Surgical Procedure*, Heart (All Fields) or Cardiac Surgical Procedure* (All Fields) or Heart Surgical Procedure* (All Fields) or Procedure*, Heart Surgical (All Fields) |
| #7 | **#5 AND #6** |

**Table S2** The search strategy for Cochrane Library

| Order | Strategy |
| --- | --- |
| #1 | MeSH descriptor: [Postoperative Cognitive Complications] 1 tree(s) exploded |
| #2 | MeSH descriptor: [Emergence Delirium] explode all trees |
| #3 | (perioperative neurocognitive disorder):ti,ab,kw |
| #4 | cognit* OR (cognition disorder) OR (cognition impairment) OR (cognition decline) OR (cognitive dysfunction) OR (cognitive function) OR delirium OR (neurocognitive disorder) OR (delayed neurocognitive recovery) OR (neurocognitive abnormalities) |
| #5 | postop* OR postoperative* OR perioperative OR (postoperative period) |
| #6 | #4 AND #5 |
| #7 | #1 OR #2 OR #3 OR #6 |
| #8 | MeSH descriptor: [Acupuncture] explode all trees |
| #9 | MeSH descriptor: [Acupuncture Therapy] explode all trees |
| #10 | MeSH descriptor: [Electroacupuncture] explode all trees |
| #11 | (Transcutaneous Electrical Acupoint Stimulation):ti,ab,kw |
| #12 | Acupuncture OR Electroacupuncture OR EA OR (Transcutaneous Electrical Acupoint Stimulation) OR TEAS |
| #13 | #8 OR #9 OR #10 OR #11 OR #12 |
| #14 | random OR randomization OR randomized OR randomised OR randomly |
| #15 | #7 AND #13 AND #14 |
| #16 | [Thoracic Surgery] explode all trees |
| #17 | [Cardiac Surgical Procedures] explode all trees |
| #18 | (Thoracic Surgery):ti,ab,kw OR (Surgery, Thoracic):ti,ab,kw OR (Surgery, Cardiac):ti,ab,kw OR (Surgery, Heart):ti,ab,kw AND (Cardiac Surgery):ti,ab,kw |
| #19 | (Procedure*, Cardiac Surgical):ti,ab,kw OR (Surgical Procedure*, Cardiac):ti,ab,kw OR (Surgical Procedure*, Heart):ti,ab,kw OR (Cardiac Surgical Procedure*):ti,ab,kw AND (Heart Surgical Procedure*):ti,ab,kw |
| #20 | #16 OR #17 OR #18 OR #19 |
| #21 | #15 AND #21 in Trials |

**Table S3** The search strategy for EMBASE

| Order | Strategy |
| --- | --- |
| #32 | #19 AND #25 AND #31 |
| #31 | #26 OR #27 OR #28 OR #29 OR #30 |
| #30 | randomly |
| #29 | randomised |
| #28 | randomized |
| #27 | randomization |
| #26 | random* |
| #25 | #20 OR #21 OR #22 OR #23 OR #24 |
| #24 | 'acupuncture Points' OR acupoint |
| #23 | 'transcutaneous electrical acupoint stimulation' |
| #22 | electroacupuncture |
| #21 | 'acupuncture Therapy' |
| #20 | acupuncture |
| #19 | #1 OR #2 OR #3 OR #18 |
| #18 | #13 AND #17 |
| #17 | #14 OR #15 OR #16 |
| #16 | 'postoperative period' |
| #15 | postoperative* |
| #14 | postop* |
| #13 | #4 OR #5 OR #6 OR #7 OR #8 OR #9 OR #10 OR #11 OR #12 |
| #12 | 'neurocognitive abnormalities' |
| #11 | 'delayed neurocognitive recovery' |
| #10 | 'neurocognitive disorder' |
| #9 | delirium |
| #8 | 'cognitive function' |
| #7 | 'cognitive dysfunction' |
| #6 | 'cognition decline' |
| #5 | 'cognition impairment' |
| #4 | 'cognition disorder' |
| #3 | 'perioperative neurocognitive disorder' |
| #2 | 'perioperative delirium' |
| #1 | 'postoperative cognitive complications' |

**Table S4** The search strategy for Scopus

| ( TITLE-ABS-KEY ( acupuncture OR electroacupuncture OR ea OR "transcutaneous electricalacupoint stimulation" OR teas ) AND TITLE-ABS-KEY ( cognit* OR "cognition disorder" OR "cognition impairment" OR " cognition decline" OR "cognitive dysfunction" OR "cognitive function" OR delirium OR "neurocognitive disorder" OR "delayed neurocognitive recovery" OR "neurocognitive abnormalities" ) AND ( thoracic* OR cardiac* OR heart* OR coronary* OR "coronary artery bypass grafting" OR "coronary artery bypass grafting" OR "valve replacement" OR valve* OR "cardiopulmonary bypass" ) AND TITLE-ABS-KEY ( random OR randomization OR randomized OR randomised OR randomly ) ) AND TITLE-ABS-KEY ( postop* OR postoperative* OR perioperative OR "postoperative period" ) |
| --- |

**Table S5** The search strategy for CNKI

| (SU=认知障碍 + 认知功能 + 谵妄 + 神经功能 + 神经认知障碍 + 神经认知) AND (SU=电针 + 经皮穴位电刺激 + 针灸 + 针刺 + 针灸疗法) AND (FT=老年 + 高龄 + 老人 + 60岁) AND (FT=术后 + 围术期) AND (FT=心脏 + 心脏手术 + 冠状动脉搭桥术 + 冠状动脉旁路移植术 + 瓣膜置换术 + 瓣膜 + 体外循环) AND (FT=随机) |
| --- |

English translation:

**Table S5** The search strategy for CNKI

| (SU= cognitive disorder + cognitive function + delirium + neurological function + neurocognitive disorder + cognition) AND (SU= electroacupuncture + transcutaneous electrical acupoint stimulation + acupuncture + needling + acupuncture therapy) AND (FT= elderly + aged + senior + 60 years old) AND (FT=postoperative + perioperative) AND (FT= heart + cardiac surgery + coronary artery bypass graft + coronary artery bypass surgery + valve replacement + valve + cardiopulmonary bypass) AND (FT= randomized) |
| --- |

**Table S6** The search strategy for Wangfang Data

| (主题=认知障碍 OR 认知功能 OR 谵妄 OR 神经功能 OR 神经认知 OR 认知) AND (主题=电针 OR 经皮穴位电刺激 OR 针灸 OR 针刺 OR 针灸疗法) AND (全部=老年 OR 高龄 OR 老人 OR 60岁) AND (全部=术后 OR 围术期) AND (全部=心脏 OR 心脏手术 OR 冠状动脉搭桥术 OR 冠状动脉旁路移植术 OR 瓣膜置换术 OR 瓣膜 OR 体外循环) AND (全部=随机) |
| --- |

English translation:

**Table S6** The search strategy for Wangfang Data

| (Subject= cognitive disorder OR cognitive function OR delirium OR neurological function OR neurocognitive OR cognition) AND (Subject= electroacupuncture OR transcutaneous electrical acupoint stimulation OR acupuncture OR needling OR acupuncture therapy) AND (All= elderly OR aged OR senior OR 60 years old) AND (All= postoperative OR perioperative) AND (All= heart OR cardiac surgery OR coronary artery bypass graft OR coronary artery bypass surgery OR valve replacement OR valve OR cardiopulmonary bypass) AND (All= randomized) |
| --- |

**Table S7** The search strategy for VIP

| U=(认知障碍 OR 认知功能 OR 谵妄 OR 神经功能 OR 神经认知 OR 认知) AND M=(电针 OR 经皮穴位电刺激 OR 针灸 OR 针刺 OR 针灸疗法) AND U=(心脏 OR 心脏手术 OR 冠状动脉搭桥术 OR 冠状动脉旁路移植术 OR 瓣膜置换术 OR 瓣膜 OR 体外循环) AND U=(随机) |
| --- |

English translation:

**Table S7** The search strategy for VIP

| U=(cognitive disorder OR cognitive function OR delirium OR neurological function OR neurocognitive OR cognition) AND M=(electroacupuncture OR transcutaneous electrical acupoint stimulation OR acupuncture OR needling OR acupuncture therapy) AND U=(heart OR cardiac surgery OR coronary artery bypass graft OR coronary artery bypass surgery OR valve replacement OR valve OR cardiopulmonary bypass) AND U=(randomized) |
| --- |

**Table S8** The search strategy for Sinomed

| ( "认知障碍"[常用字段:智能] OR "认知功能 "[常用字段:智能] OR "谵妄"[常用字段:智能] OR "神经功能"[常用字段:智能] OR "神经认知"[常用字段:智能] OR "认知"[常用字段:智能]) AND( "电针"[常用字段:智能] OR "经皮穴位电刺激"[常用字段:智能] OR "针灸"[常用字段:智能] OR "针刺"[常用字段:智能] OR "针灸疗法"[常用字段:智能]) AND( "术后"[常用字段:智能] OR "围术期"[常用字段:智能]) AND "随机"[全部字段:智能] AND( "心脏"[全部字段:智能] OR "心脏手术"[全部字段:智能] OR "冠状动脉搭桥术"[全部字段:智能] OR "冠状动脉旁路移植术"[全部字段:智能] OR "瓣膜置换术"[全部字段:智能] OR "瓣膜"[全部字段:智能] OR "体外循环"[全部字段:智能]) |
| --- |

English translation:

**Table S8** The search strategy for Sinomed

| ("cognitive disorder"[Common Field: Intelligence] OR "cognitive function"[Common Field: Intelligence] OR "delirium"[Common Field: Intelligence] OR "neurological function"[Common Field: Intelligence] OR "neurocognitive"[Common Field: Intelligence] OR "cognition"[Common Field: Intelligence] ) AND( "electroacupuncture"[Common Field: Intelligence] OR "transcutaneous electrical acupoint stimulation"[Common Field: Intelligence] OR "acupuncture"[Common Field: Intelligence] OR "needling"[Common Field: Intelligence] OR "acupuncture therapy"[Common Field: Intelligence]) AND( "postoperative"[Common Field: Intelligence] OR "perioperative"[Common Field: Intelligence]) AND "randomized"[All Fields: Intelligence] AND( "heart"[All Fields: Intelligence] OR "cardiac surgery"[All Fields: Intelligence] OR "coronary artery bypass graft"[All Fields: Intelligence] OR "coronary artery bypass surgery"[All Fields: Intelligence] OR "valve replacement"[All Fields: Intelligence] OR "valve"[All Fields: Intelligence] OR "cardiopulmonary bypass"[All Fields: Intelligence]) |
| --- |

**Table S9** The search strategy for Registers (http://www.chictr.org.cn/)

| **Disease =** (认知障碍 OR 认知功能 OR 术后谵妄 OR 神经功能 OR 神经认知 OR 认知) |
| --- |
| Intervention **=** (电针 OR 经皮穴位电刺激 OR 针刺**)** |

English translation:

**Table S9** The search strategy for Registers (http://www.chictr.org.cn/)

| **Disease =** (cognitive disorder OR cognitive function OR postoperative delirium OR neurological function OR neurocognitive OR cognition) |
| --- |
| Intervention **=** (electroacupuncture OR transcutaneous electrical acupoint stimulation OR acupuncture**)** |

**Table S10** The search strategy for Registers (ClinicalTrials.gov)

| **Disease = (**cognit* OR "cognition disorder" OR "cognition impairment" OR "cognition decline" OR "cognitive dysfunction" OR "cognitive function" OR "delirium" OR "neurocognitive disorder" OR "delayed neurocognitive recovery" OR "neurocognitive abnormalities") |
| --- |
| Intervention **= (**acupuncture OR electroacupuncture OR "EA" OR "transcutaneous electrical acupoint stimulation" OR "TEAS") |
